# Angiotensin II type 2 receptor antagonist reduces bleomycin-induced pulmonary fibrosis in mice

**DOI:** 10.1186/1465-9921-9-43

**Published:** 2008-05-23

**Authors:** Yuko Waseda, Masahide Yasui, Yoriko Nishizawa, Kanako Inuzuka, Hazuki Takato, Yukari Ichikawa, Atsuro Tagami, Masaki Fujimura, Shinji Nakao

**Affiliations:** 1 Respiratory Medicine, Cellular Transplantation Biology, Kanazawa University Graduate School of Medical Science, 13-1 Takara-machi, Kanazawa 920-8641, Japan

## Abstract

**Background:**

The role of angiotensin II type 2 receptor (AT2) in pulmonary fibrosis is unknown. To evaluate the influence of angiotensin II type 1 receptor (AT1) and AT2 antagonists in a mouse model of bleomycin (BLM)-induced pulmonary fibrosis.

**Methods:**

We examined effects of the AT1 antagonist (AT1A) olmesartan medoxomil (olmesartan) and the AT2 antagonist (AT2A) PD-123319 on BLM-induced pulmonary fibrosis, which was evaluated by Ashcroft's pathological scoring and hydroxyproline content of lungs. We also analyzed the cellular composition and cytokine levels in bronchoalveolar lavage fluid (BALF).

**Results:**

With olmesartan, the lung fibrosis score and hydroxyproline level were significantly reduced, and lymphocyte and neutrophil counts and tumor necrosis factor (TNF)-α levels in BALF were reduced on day 7. On day 14, macrophage and lymphocyte counts in BALF were reduced, accompanied by a reduction in the level of transforming growth factor (TGF)-β_1_. With PD-123319, the lung fibrosis score and hydroxyproline level were reduced. On day 7, macrophage, lymphocyte, and neutrophil counts in BALF were reduced, accompanied by reductions in TNF-α and monocyte chemoattractant protein (MCP)-1 levels. On day 14, macrophage, lymphocyte, and neutrophil counts in BALF were also reduced, accompanied by a reduction in the level of macrophage inflammatory protein (MIP)-2 level but not TGF-β_1_.

**Conclusion:**

Both AT1 and AT2 are involved in promoting interstitial pneumonia and pulmonary fibrosis via different mechanisms of action.

## Background

It is known that the renin-angiotensin system (RAS) has a variety of actions in vivo. A classical action of this system includes contraction of the blood vessels and increase in intravascular volume, both of which are involved in elevation of blood pressure. The RAS is active not only in the vascular system but also in various tissues.

Renin converts angiotensinogen into angiotensin I (AI), and angiotensin-converting enzymes (ACE) convert AI into angiotensin II (AII). AII is the center of activity of the RAS. To date, four angiotensin receptors, angiotensin type 1 receptor (AT1), type 2 receptor (AT2), type 3 receptor (AT3), and type 4 receptor (AT4), have been identified [1], and AT1 has further been subdivided into AT1a and AT1b [2]. AII binds to both AT1 and AT2. Angiotensin receptor blockers (ARBs), which are often used as depressor drugs, are selective AT1 antagonists (AT1As) [3].

Recent studies have shown that AT1 is involved in fibrosis of organs. It is widely accepted that inhibition of AT1 can suppress fibrosis of the heart and kidneys [4-6]. With respect to AT1 and lung fibrosis, four studies [2,7-9] of an animal model of bleomycin (BLM)-induced pulmonary fibrosis revealed that treatment with an AT1A suppressed pulmonary hydroxyproline levels, and, therefore, AT1As can suppress pulmonary fibrosis. Of those, two studies [7,8] also revealed that AT1As suppress the transforming growth factor (TGF)-β_1 _level in bronchoalveolar lavage fluid (BALF) simultaneously.

Although all studies [10,11] of AT2 in the heart indicated that stimulation of AT2 could suppress fibrosis, the role of AT2 in fibrosis of the kidney is controversial. One study [12] found that stimulation of AT2 suppressed fibrosis, and the authors [10-12] supposed that the inhibitory effect of AT1A is through the relative stimulation of AT2, whereas other studies [13,14] showed that inhibition of AT2 suppressed fibrosis of the kidney.

The role of AT2 in pulmonary fibrosis is not well known and has been reported only by Melanie, et al. [15]. These authors found that AII signaling occurred primarily via AT1 in normal fibroblasts, while AT2-mediated effects were dominant on activated fibroblasts. In the present study, we examined the involvement of AT2 in pulmonary fibrosis by evaluating the influence of AT1A and AT2A in a mouse model of BLM-induced pulmonary fibrosis, with the goal of clarifying the differences in the roles of AT1 and AT2 in pulmonary fibrosis.

## Methods

### Animal preparation of BLM-induced pulmonary fibrosis

Adult male 8-wk ICR mice were purchased from Sankyo Laboratories (Tokyo, Japan). All mice were maintained under standard conditions with free access to water and rodent laboratory food. Mice were anesthetized by inhalation of ether, and BLM (Nippon Kayaku, Tokyo, Japan) dissolved in 100 μl saline solution was administered intratracheally at a dose of 2.0 mg/kg body weight. From the same day of BLM administration, the AT1-specific antagonist olmesartan medoxomil (olmesartan; Daiichi Sankyo, Tokyo, Japan) in 200 μl of 0.5% carboxymethyl cellulose was administered orally at a dose of 0.1 or 1 mg/kg/day for 14 sequential days. The AT2-specific antagonist PD123319 (R&D Systems, Minneapolis, MN, USA), dissolved in 200 μl saline solution, was administered subcutaneously at a dose of 0.5 or 5 mg/kg/day with 2-week mini-osmotic pumps (Alzet Model 2002; Alza, Palo Alto, CA, USA) for 14 sequential days. All animal procedures in this study complied with the standards set out in the Guidelines for the Care and Use of Laboratory Animals of the Takara-machi Campus of Kanazawa University.

### Histopathological evaluation

Mice were anesthetized by intraperitoneal injection of pentobarbitone sodium (60 mg/kg body wt; Boehringer Ingelheim, Bilberach, Germany) and killed on day 14. The left lung was fixed at a transpulmonary pressures of 25 cmH_2_O with 10% formaldehyde neutral buffer solution for at least 48 h and then embedded in paraffin. Sequential 3 μm sections were stained with hematoxylin-eosin and Azan-Mallory stains. Severity of fibrosis was semi-quantitatively assessed according to the method of Ashcroft et al. [16]. The lung fibrosis score was expressed as a mean grade of fibrosis for each sample.

### Measurement of lung hydroxyproline content

Mice from each group were killed on day 14, and the lungs were removed. Frozen lung tissues were homogenized by a polytron tissue homogenizer in saline containing 0.1 M phenylmethylsulfonylfluoride. The homogenized sample was hydrolyzed in 6 N HCl, and the hydroxyproline concentration was determined according to the method of Schrier et al. [17].

### Bronchoalveolar Lavage (BAL)

Mice from each group were killed on day 7 and 14, and BAL was performed. After excision of the trachea, a plastic cannula was inserted into the trachea, and 2 ml saline solution was injected gently with a syringe and then withdrawn. This procedure was repeated three times. A 200 μl aliquot of BAL fluid (BALF) was reserved for total cell counts and evaluation of cell differentiation. The remaining BALF was centrifuged immediately at 1,100 rpm for 10 min. Total cell number was determined with a standard hemocytometer. Cell differentiation was examined by counting at least 200 cells on a smear prepared with cytospin and Wright-Giemsa staining. Supernatants were stored at -80°C until used for measurement of cytokines.

Tumor necrosis factor (TNF)-α, macrophage inflammatory protein (MIP)-1α, MIP-2, monocyte chemoattractant protein (MCP)-1 and TGF-β_1 _levels in BALF supernatants were measured by enzyme-linked immunosorbent assay (ELISA) (Quantikine; R&D Systems). For the measurement of TGF-β_1_, BALF samples underwent acidification to convert the latent TGF-β_1 _to the active form. 1N HCl was used followed by neutralization with 1N NaOH. Total (latent and active form) TGF-β_1 _levels were measured.

### Quantitative Reverse Transcriptase Polymerase Chain Reaction analysis (RT-PCR)

Lungs were harvested on day 7 and 14 after bleomycin administration, and total RNA was isolated from frozen lung specimens using a RNeasy Mini Kit according to the manufacurer's instructions (Qiagen, Hilden, Germany). RNA yield and purity were determined by spectrophotometry. RNA was then reverse transcribed into cDNA and amplified using the Reverse Transcription System (Promega, Madison, WI). Real-time RT-PCR was performed using fluorogenic SYBR Green using a LightCycler thermal cycler system according to the manufacturer's instructions (Roche Diagnostics GmbH, Mannheim, Germany). Seven mice of each genotype were examined. The primers for AT1a, AT1b, AT2, and glyceraldehydes-3-phasphate dehydrogenase (GAPDH) were designed according to previous studies and were synthesized by Takara Bio Co. (Tokyo, Japan). The sense and antisense primers used were as follows: AT1a primer, 5'-GGA CAC TGC CAT GCC CAT AAC-3' and 5'-TGA GTG CGA CTT GGC CTT TG-3'; AT1b primer, 5'-CTG CTA TGC CCA TCA CCA TCT G-3' and 5'-GAT AAC CCT GCA TGC GAC CTG-3'; AT2 primer, 5'-CTC CAG GTT TAG ACT GCT GCC TTC-3' and 5'-GGT TGA CAC CGA GTT TGT CAT TTG-3'; and GAPDH primer, 5'-TGT GTC CGT CGT GGA TCT GA-3' and 5'-TTG CTG TTG AAG TCG CAG GAG-3'.

### Experiment 1 (Effect of olmesartan)

**Figure 1:**
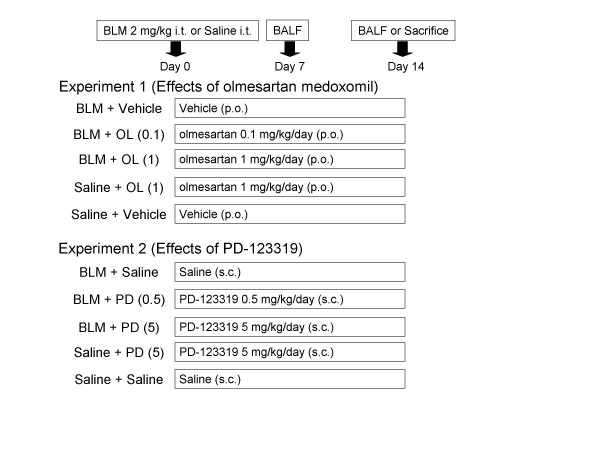
**Study protocol**. *Arrows *indicate times of death. i.t.; intratracheal injection, p.o.; per os, s.c.; subcutaneous injection, BLM; bleomycin, OL; olmesartan medoxomil, PD; PD-123319.

As shown in Figure 1, the animals were divided into five groups: the uncombined BLM i.t. group, two groups treated with a combination of BLM and an olmesartan at dose levels of 0.1 or 1 mg/kg p.o., the uncombined olmesartan treatment group, and the control group.

### Experiment 2 (Effect of PD-123319)

As shown in Figure 1, the animals were divided into five groups: the uncombined BLM i.t. group, two groups treated with a combination of BLM and an PD-123319 at dose levels of 0.5 or 5 mg/kg s.c., the uncombined PD-123319 group, and the control group.

### Statistical analysis

Data are expressed as mean ± standard error of the mean (SEM). Statistical analysis was done with Stat View-J IV software (Brainpower, Inc., Calabasas, CA) on a Macintosh computer (Apple, Inc. Cupertino, CA). One-way analysis of variance (ANOVA), followed by Fisher's Protected Least Significant Difference (PLSD), was used to detect differences among groups, and a value of p < 0.05 was considered significant.

## Results

### Pathological score and hydroxyproline content

**Figure 2:**
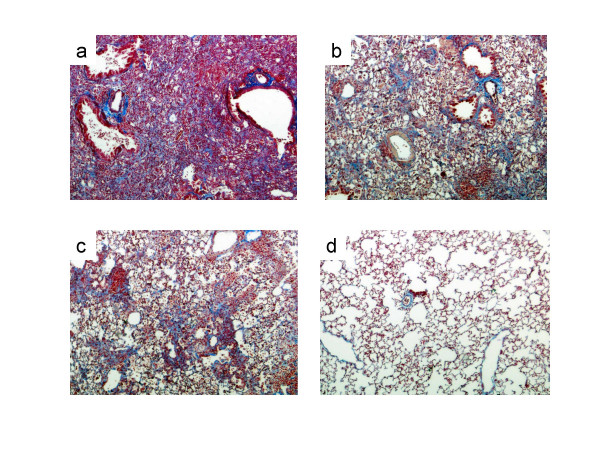
**Histologic sections of the left lung 14 days after the last dose of bleomycin (BLM)**. (a) BLM group. Pulmonary fibrosis with definite damage to the lung structure is visible. (b) BLM + olmesartan medoxomil 1 mg/kg group. Increased fibrosis with definite damage to the lung structure and formation of small fibrous masses were partially observed. (c) BLM + PD 5 mg/kg group. Increased fibrosis with definite damage to the lung structure and formation of small fibrous masses were partially observed. (d) Control group. The normal alveolar structure is visible. (Azan-Mallory × 40)

**Figure 3:**
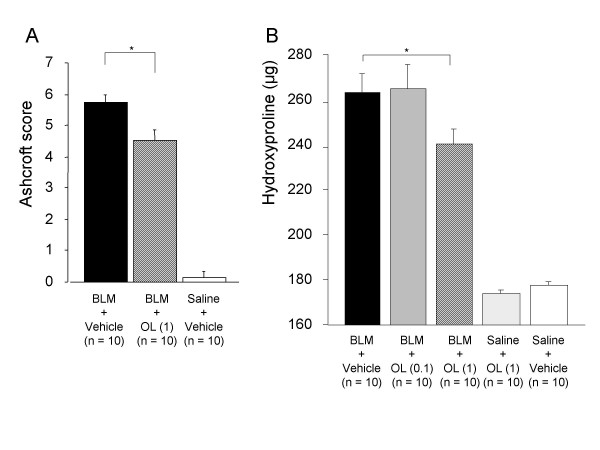
**Effect of olmesartan medoxomil on pathologic score (A) and hydroxyproline content (B)**. BLM; bleomycin, OL (0.1); olmesartan medoxomil 0.1 mg/kg, OL (1); olmesartan medoxomil 1 mg/kg. *p < 0.05.

**Figure 4:**
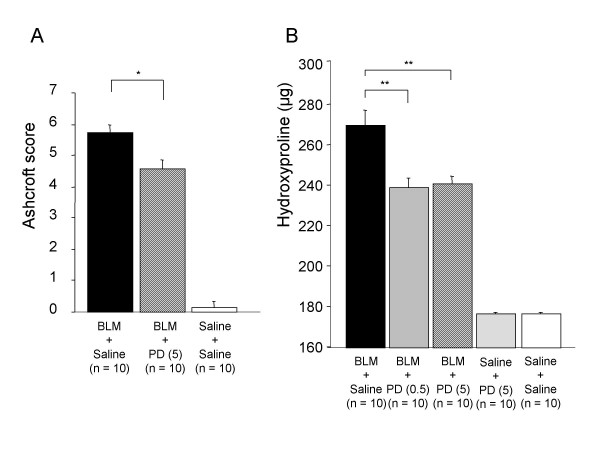
**Effect of PD-123319 on pathologic score (A) and hydroxyproline content (B)**. BLM; bleomycin, PD (0.5); PD-123319 0.5 mg/kg, PD (5); PD-123319 5 mg/kg. *p < 0.05; **p < 0.01.

Ashcroft scores for BLM-induced pulmonary fibrosis were significantly lower in the olmesartan treatment groups (Figure 2b, Figure 3A) and in the PD-123319 treatment groups (Figure 2c, Figure 4A) than in the vehicle treatment groups.

Hydroxyproline levels in lungs of mice with BLM-induced pulmonary fibrosis were significantly lower in mice treated with 1 mg/kg of olmesartan (Figure 3B) as they were with 0.5 and 5 mg/kg of PD-123319 (Figure 4B) than in the control groups.

### Cell components and cytokine levels in BALF

#### Effect of olmesartan

On day 7, total cell, lymphocyte, and neutrophil counts were lower in the mice treated with both BLM and 1 mg/kg olmesartan than in the group treated with BLM alone (Table 1). TNF-α levels were also significantly lower in the BLM and olmesartan treatment group than in the BLM treatment group (Table 2). No significant intergroup difference was noted in the level of any other cytokines or chemokines. MIP-1α and MIP-2 levels were below the limits of detection in all groups.

**Table 1:** Effect of Olmesartan Medoxomil on cell components in bronchoalveolar lavage fluid

			Cell differentiation (× 10^5^/ml)		
		Total cell number (× 10^5^/ml)	Mac	Lym	Neu
Day 7					
	BLM (n = 8)	182.4 ± 7.1	67.6 ± 4.0	99.9 ± 5.6	14.9 ± 3.0
	BLM + OL (n = 8)	127.8 ± 13.4 **	55.9 ± 6.2	64.5 ± 7.8 **	7.4 ± 1.2 *
	OL (n = 8)	7.8 ± 2.5	6.4 ± 2.1	1.0 ± 0.3	0.4 ± 0.3
Day 14					
	BLM (n = 8)	163.7 ± 15.6	87.6 ± 8.1	61.9 ± 10.0	2.5 ± 0.8
	BLM + OL (n = 8)	108.5 ± 34.3 *	52.6 ± 16.6 **	37.6 ± 16.8 *	1.5 ± 0.7
	OL (n = 8)	15.4 ± 2.6	12.9 ± 3.0	0.2 ± 0.1	0.1 ± 0.1

Mac; Macrophages, Lym; lymphocytes, Neu; neutrophils, BLM; bleomycin, OL; olmesartan medoxomil

Values are expressed as mean ± SEM.

*p < 0.05, **p < 0.01 as compared to the BLM group.

**Table 2:** Effect of Olmesartan Medoxomil on cytokine levels in bronchoalveolar lavage fluid

Day 7		TNF-α (pg/ml)	MCP-1 (pg/ml)	TGF-β_1 _(pg/ml)
	BLM (n = 8)	20.7 ± 1.7	766.3 ± 100.6	331.3 ± 17.7
	BLM + OL (n = 8)	15.6 ± 1.6 *	701.3 ± 97.6	318.3 ± 29.9
	OL (n = 8)	5.1 ± 0.0	2.0 ± 0.0	19.9 ± 3.4
Day 14		MIP-2 (pg/ml)	TGF-β_1 _(pg/ml)	
	BLM (n = 8)	7.1 ± 2.4	287.9 ± 35.2	
	BLM + OL (n = 8)	1.5 ± 0	173.4 ± 49.9 *	
	OL (n = 8)	1.5 ± 0	21.2 ± 7.3	

BLM; bleomycin, OL; olmesartan medoxomil

Values are expressed as mean ± SEM.

*p < 0.05 as compared to the BLM group.

On day 14, total cell, macrophage, and lymphocyte counts were significantly lower in the group treated with both BLM and 1 mg/kg olmesartan than the group treated with BLM alone (Table 1). TGF-β_1 _levels were also significantly lower in the group treated with both BLM and olmesartan than in the group treated with BLM alone (Table 2). No significant intergroup difference was noted in the level of any other cytokines or chemokines. TNF-α, MIP-1α, and MCP-1 levels were below the limits of detection in all groups.

#### Effect of PD-123319

On day 7, total cell, macrophage, lymphocyte, and neutrophil counts were significantly lower in the group treated with both BLM and 5 mg/kg PD-123319 than in the group treated with BLM alone (Table 3). TNF-α and MCP-1 levels were also significantly lower in the group treated with both BLM and PD-123319 than in the group treated with BLM alone (Table 4). There was no significant intergroup difference in TGF-β_1 _level. MIP-1α and MIP-2 levels were below the limits of detection in all groups.

**Table 3:** Effect of PD-123319 on cell components in bronchoalveolar lavage fluid

			Cell differentiation (× 10^5^/ml)		
		Total cell number (× 10^5^/ml)	Mac	Lym	Neu
Day 7					
	BLM (n = 8)	183.3 ± 7.9	69.0 ± 4.2	99.1 ± 6.1	15.3 ± 3.4
	BLM + PD (n = 8)	118.9 ± 6.1 *	52.8 ± 4.1 *	57.7 ± 3.2 ††	6.6 ± 1.4 **
	PD (n = 8)	8.0 ± 3.1	6.5 ± 2.6	1.0 ± 0.3	0.5 ± 0.3
Day 14					
	BLM (n = 8)	151.8 ± 20.8	57.1 ± 7.6	86.6 ± 14.2	7.9 ± 2.0
	BLM + PD (n = 8)	84.1 ± 17.1 †	30.5 ± 3.2 **	51.8 ± 15.5 *	1.8 ± 0.5 *
	PD (n = 8)	8.7 ± 1.3	6.8 ± 0.9	1.3 ± 0.2	0.6 ± 0.3

Mac; Macrophages, Lym; lymphocytes, Neu; neutrophils, BLM; bleomycin, PD; PD-123319

Values are expressed as mean ± SEM.

*p < 0.05, **p < 0.01, †p < 0.005, ††p < 0.001 as compared to the BLM group.

**Table 4:** Effect of PD-123319 on cytokine levels in bronchoalveolar lavage fluid

Day 7		TNF-α (pg/ml)	MCP-1 (pg/ml)	TGF-β_1 _(pg/ml)
	BLM (n = 8)	21.0 ± 1.9	795.6 ± 109.2	334.8 ± 19.7
	BLM + PD (n = 8)	13.9 ± 2.6 *	441.8 ± 85.9 *	374.7 ± 46.4
	PD (n = 8)	5.1 ± 0.0	2.1 ± 0.1	20.7 ± 4.1
Day 14		MIP-2 (pg/ml)	TGF-β_1 _(pg/ml)	
	BLM (n = 8)	7.0 ± 1.1	390.0 ± 46.5	
	BLM + PD (n = 8)	1.5 ± 0 *	406.1 ± 25.9	
	PD (n = 8)	1.5 ± 0	18.0 ± 3.3	

BLM; bleomycin, PD; PD-123319

Values are expressed as mean ± SEM.

*p < 0.05 as compared to the BLM group.

On day 14, total cell, macrophage, lymphocyte, and neutrophil counts were significantly lower in the group treated with both BLM and 5 mg/kg PD-123319 than in the group treated with BLM alone (Table 3). The MIP-2 level was also significantly lower in the group treated with both BLM and PD-123319 than in the group treated with BLM alone. There was no significant intergroup difference in TGF-β_1 _level (Table 4). TNF-α, MIP-1α, and MCP-1 levels were below the limits of detection in all groups.

### AT1 and AT2 Expression in the lung

**Figure 5:**
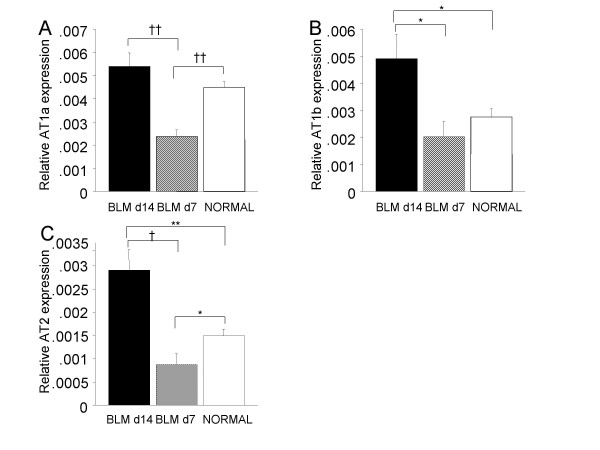
**Relative expression levels of AT1a (A), AT1b (B) and AT2 (C) mRNA as determined by real-time PCR**. BLM; bleomycin. *p < 0.05; **p < 0.01; †p < 0.005; ††p < 0.001.

As shown in Figure 5, AT1a expression was significantly lower on day 7 than day 0, and higher on day 14 than day 7. It had inclination to be higher on day 14 than day 0, but, did not have a significant difference. AT1b expression was not significantly different on day 7 and day 0. But it was significantly higher on day 14 than day 7 and day 0. AT2 expression was significantly lower on day 7 than day 0, and was significantly higher on day 14 than day 7 and day 0.

## Discussion

In this study, we showed expressions of AT1a, AT1b and, AT2 in the lungs of a BLM-induced model of pulmonary fibrosis. On day 14 with BLM treatment, lungs up-regulated AT1b and AT2 compared with on day 0, and AT1a, AT1b and AT2 compared with on day 7. It has been reported that lungs up-regulated AT1 and AT2 on day 14 [18], which is consistent with our results. Of note, only mice and other rodents have these duplicated AT1 genes [19,20], whereas other mammals studied thus far have a single AT1 gene [21]. It has been reported that blood pressure of both AT1a and AT1b knockout mice had lower than levels of only AT1a knockout mice, and as low as levels of angiotensinogen knockout mice. So both isoform may have biologically activation. The difference of action between AT1a and AT1b in lung was not known, but both of them were expressed in lungs and may be concerned in lung fibrosis. Therefore, the inhibition of AT1 and AT2, which are increasing in lungs, may suppress lung fibrosis.

In the BLM-induced model of pulmonary fibrosis, inflammatory cytokines such as TNF-α increase immediately after BLM administration, and subsequently, levels of chemokines such as MCP-1, MIP-1α, and MIP-2 increase, resulting in the infiltration of inflamed cells into the lungs. When alveolitis occurs, levels of growth factors such as TGF-β_1_, platelet-derived growth factor (PDGF), and insulin-like growth factor (IGF)-1 increase, and pulmonary fibrosis gradually develops [22-24]. In the present study, both AT1A and AT2A inhibited a variety of BLM-induced pulmonary fibrosis processes. Furthermore, we found that the inhibitory effect of AT1A on BLM-induced pulmonary fibrosis is not mediated by AT2.

It has been reported that AT1A has an inhibitory effect on pulmonary fibrosis in both the BLM- and amiodarone-induced pulmonary fibrosis models [2,7-9,25,26]. Most reports suggest that pulmonary fibrosis is inhibited by AT1A, while the inhibitory effect was not investigated in the study by Keogh et al. [26]. The present findings support the idea that AT1A inhibits pulmonary fibrosis.

AT1A has been reported to inhibit fibrosis in heart and kidney fibrosis models. It is believed that AT1A has a fibrosis inhibitory effect, regardless of the organ and tissue [4-6]. In cardiac fibroblast cells [27], vascular smooth muscle cells [28], bronchial smooth muscle cells [29], and kidney mesangial cells [30] cultured with AII in vitro, increased levels of TGF-β_1 _were observed [8]. In addition, stimulation of AT1 in a mesangial cell has been reported to activate NF-κB [31], and therefore, AT1 is also believed to promote expression of inflammatory cytokines and chemokines. In the present study, AT1A inhibited increases in TNF-α and TGF-β_1 _levels after BLM administration. In other words, it is believed that stimulation of AT1 by AII promotes expression of these cytokines in the lung after BLM administration. In addition, Otsuka et al. [8] reported that AT1 in rat lungs was expressed in alveolar macrophages, type II alveolar epithelial cells, vascular smooth muscle cells, endothelial cells, and fibroblasts. AT1 is expressed in the alveolar epithelium, stroma cells under the respiratory tract epithelium, vessel smooth muscle and alveolar macrophages, and even in human lungs [32]. Therefore, AT1A may act in a variety of manners in the various cells of the lungs that are involved in the formation of BLM-induced fibrosis.

In cardiac fibrosis, stimulation of AT2 has been reported to inhibit fibrosis, however, the inhibitory action on renal fibrosis is controversial [12-14]. In the present study, we examined the connection between AT2 and pulmonary fibrosis, and the results clearly showed that BLM-induced pulmonary fibrosis was inhibited by AT2 antagonism. Therefore, AT2 stimulation appears to promote BLM-induced pulmonary fibrosis. These conflicting findings suggest that the role of AT2 in fibrosis may be organ- or tissue-specific.

Stimulation of AT2 as well as AT1 activates NF-κB in mesangial cells [21]. In the present study, AT2A inhibited increases in TNF-α, MCP-1, and MIP-2 after BLM administration and prevented infiltration of inflamed cells. However, there was no difference in expression of TGF-β_1_. This is in contrast to our finding that AT1A inhibited the increase in TGF-β_1 _while the inhibition of chemokines was mild. Taken together, these findings suggest that AT1 and AT2 are involved in expression of TGF-β_1 _and chemokines, respectively, in our BLM-induced pulmonary fibrosis model. Therefore, AT1 and AT2 may promote pulmonary fibrosis through different mechanisms of action.

AT2 is expressed in the type II epithelial cells in rat lung [33,34] and in human lung [31,35]. However, the details are still unclear. Differences in the mechanisms of action of AT1A and AT2A may be due to expression cells involved in the fibrosis process.

With respect to AT1A and AT2A in the heart, although AT1A decreases stroma fibrosis in a wild mouse model of myocardial infarction, the improvement brought about by AT1A is less significant in the AT2-knockout mice [10]. In a rat model of cardiac hypertrophy, fibrosis improved after AT1A administration, but this improvement disappeared when AT2A was administered simultaneously [11]. Similar findings were obtained in a mouse model of kidney fibrosis where AT1A inhibited stromal fibrosis, but the inhibition disappeared when AT2A was administered simultaneously [12]. Therefore, in addition to inhibiting the stimulation of AT1 by AII, the inhibitory effect of AT1A on fibrosis occurs through stimulation of AT2 by AII. However, in some models of kidney fibrosis, the combination of AT1A and AT2A has been reported to yield a stronger inhibition of fibrosis [13,14]. In the present study, AT1A and AT2A inhibited BLM-induced pulmonary fibrosis independently. As a result, it is expected that AT2A combined with AT1A will show greater inhibition of BLM-induced pulmonary fibrosis than either agent alone. Further testing is necessary to confirm this hypothesis.

## Conclusion

Our present analyses of a mouse model of BLM-induced pulmonary fibrosis indicate that AT1 and AT2 are involved in interstitial pneumonia and pulmonary fibrosis, and each receptor has a different mechanism of action. In the future, it will be important to examine both temporal and spatial expression of AT1 and AT2 in lung tissue in a BLM-induced pulmonary fibrosis model and in various types of pulmonary fibrosis in humans, as typified by idiopathic pulmonary fibrosis.

## Authors' contributions

YW planned and performed the experiment, conducted animal, biochemical, and histological studies, and drafted the manuscript. MY proposed and planned the experiment. YN, KI, HT, YI, and AT helped coordinate the experiment and assisted with various studies. MF planned the experiment. SN coordinated the research group. All authors read and approved the final manuscript.
